# Study of pedestrian behavior and its effective factors in Sahand new city

**Published:** 2019-08

**Authors:** Mehrdad Ghahramani, Maryam Fathi Shayan, Soghra Nazemi, Mostafa Farah Bakhsh, Mahin Dahim, Homayoun Sadeghi- Bazargani, Seyyed Rasoul Hashemi Aghdam

**Affiliations:** ^*a*^Sahand Health Complex, Tabriz University of Medical Sciences, Tabriz, Iran.; ^*b*^Oskou Health Center, Tabriz University of Medical Sciences, Tabriz, Iran.; ^*c*^Sahand Health Complex, Tabriz University of Medical Sciences, Tabriz, Iran.; ^*d*^Road Traffic Injury Research center, Tabriz University of Medical Sciences, Tabriz, Iran.; ^*e*^Health Deputy, Tabriz University of Medical Sciences, Tabriz, Iran.

## Abstract

**Background:**

This research is a descriptive-analytical research whose general purpose is to determine the traffic behavior of pedestrians and the factors affecting it in Sahand city.

**Methods:**

The information of this project was collected through questionnaires provided by more than 200 pedestrians of Sahand city. In order to achieve a total traffic score for the traffic behavior of pedestrians the mean of the total scores of the questionnaires are calculated. The average obtained indicates that Sahand pedestrians receive an average of 73 from100 in traffic behavior. Also, the relationship between the demographic characteristics (age, gender, education, and marriage) and the pedestrian score was studied. In studying the impact of gender, the obtained results show that females score 74.9 have a safer behavior than males with a score of 70.8. In studying the age, it is obtained that the most unsafe scores belong to the group of 12 to 24 years with a score of 66.4 and 56 to 80 years with a score of 70.5. The 24-40 years with score of 74 and 41-55 with score of 76 are the safest age groups of Sahand pedestrians. In studying the marriage, we find that married pedestrians receive higher scores (74) than single pedestrians (65). In studying the education level, it can be seen that the lowest traffic score is for illiterate pedestrians with a rating of 61.4 and up to 6 literacy classes with a score of 70.5 and the highest average is for pedestrians with a bachelor's literacy (79.4) and Masters (83.3). The statistical results were analyzed using SPSS software package.

**Results:**

The coefficient of Cronbach 's alpha test, was 0.759 and showed that all questions had good reliability. The Pearson correlation coefficient between age and traffic scores was 0.152 and indicated a weak and positive correlation which the Sig level showed that the relationship was significant. Spearman 's correlation coefficient for education and traffic score was 0.327 and the Sig was also less than 0.05 indicating a moderate and significant correlation. According the non-normality of the gender variables, the Sig value of 0.072, Mann-Whitney test indicates no significant difference between the women and men in the traffic score. In addition, according to non- normality of single and married groups, the Sig value of Mann-Whitney test 0.000 indicates that the two samples are statistically significantly different and married people is better than single ones in traffic behavior.

**Conclusions:**

According to results, maturity and experience which comes from increasing education level, marriage, increasing age will have a proper effect on traffic behavior of Sahand pedestrians. But regarding gender, it was observed that there was no significant relationship between gender and traffic behavior of Sahand pedestrians.

**Keywords:**

Pedestrian behavior, Safe community, Demographic characteristics, Sahand new city

